# Dual-Element Transducer with Phase-Inversion for Wide Depth of Field in High-Frequency Ultrasound Imaging

**DOI:** 10.3390/s140814278

**Published:** 2014-08-05

**Authors:** Jong Seob Jeong

**Affiliations:** Department of Medical Biotechnology, Dongguk University, Seoul 100-715, Korea; E-Mail: jjsspace@dongguk.edu; Tel./Fax: +82-2-2260-3309

**Keywords:** high frequency ultrasound imaging, depth of field, signal-to-noise ratio, dual-element transducer, phase-inversion, multi-focal zone

## Abstract

In high frequency ultrasound imaging (HFUI), the quality of focusing is deeply related to the length of the depth of field (DOF). In this paper, a phase-inversion technique implemented by a dual-element transducer is proposed to enlarge the DOF. The performance of the proposed method was numerically demonstrated by using the ultrasound simulation program called Field-II. A simulated dual-element transducer was composed of a disc- and an annular-type elements, and its aperture was concavely shaped to have a confocal point at 6 mm. The area of each element was identical in order to provide same intensity at the focal point. The outer diameters of the inner and the outer elements were 2.1 mm and 3 mm, respectively. The center frequency of each element was 40 MHz and the f-number (focal depth/aperture size) was two. When two input signals with 0° and 180° phases were applied to inner and outer elements simultaneously, a multi-focal zone was generated in the axial direction. The total −6 dB DOF, *i.e.*, sum of two −6 dB DOFs in the near and far field lobes, was 40% longer than that of the conventional single element transducer. The signal to noise ratio (SNR) was increased by about two times, especially in the far field. The point and cyst phantom simulation were conducted and their results were identical to that of the beam pattern simulation. Thus, the proposed scheme may be a potential method to improve the DOF and SNR in HFUI.

## 1. Introduction

In recent years, high-frequency ultrasound imaging (HFUI) capable of providing high spatial resolution has been applied to ophthalmology, skin, and small animal experiments [1–4]. However, HFUI is suffered from high attenuation and short depth of field (DOF) because attenuation and DOF are proportional to the frequency and wavelength, respectively. Typically, a long DOF is desirable because good focusing ability can be maintained within the DOF range resulting in the good quality of ultrasound image. Additionally, a short DOF is one of the reasons for reducing signal-to-noise ratio (SNR) in the far field [5–7]. In general, the length of DOF can be extended by increasing the focal depth with the limited aperture size, or decreasing the aperture size with the fixed focal depth while the intensity at the focal zone is reduced in both cases.

In order to solve the aforementioned problems, several researchers have been proposed techniques to increase DOF. One method is using the specially designed axicon lens capable of enlarging DOF [8–10]. However, typically, a lens decreases the intensity of the transmitted and received ultrasound. The other method is the multi-transmit focusing capable of generating multi-focal points using an array transducer [11]. Synthetic focusing method using an annular array is another approach to increase DOF [12,13]. However, array transducers including annular array transducer require a complicated fabrication process and a more sophisticated system.

Along the same vein to extend DOF without the array transducers, in this study, the phase-inversion technique by using the dual-element transducer is presented. The proposed method can generate a multi-focal zone in the axial direction by activating the inner disc and outer annular element with phase inverted signals at the same time. A numerical ultrasound simulation program (Field-II) [14–16] was employed to implement transmit/receive beam pattern, point target, and cyst target simulations. The performance of the proposed method was compared with the single element transducer.

## 2. Methods

### 2.1. Phase-Inverted Excitation

The phase-inverted excitation has been applied to the optics for producing the multi-radial beams resulting in generation of the coaxial-focal shift, *i.e.*, multi-focal zone. It can be implemented by adjusting a phase mask or apodizer [17–19] based on phase control. The first focal zone is shifted in the second one by changing the phase of each portion. The normalized amplitude distribution in the N portion annular phase-shifting apodizer can be described by the below Equation (1) [17].

(1) $$G(\rho ,u)=2\sum_{j=1}^{N}\exp(i\phi_{j})\int_{r_{j-1}}^{r_{j}}rJ_{0}(\rho r)\times \exp\left[-\left(\frac{1}{w^{2}}+\frac{\text{iu}}{2}\right)r^{2}\right]\text{d}r$$

where *N* is the number of portion, *ϕ_j_* is the phase shift of the portion *j*, *r* is the radial coordinate of the objective aperture, *ρ* = (2*π*/*λ*)(*NA*)*R* and *u* = (2*π*/*λ*)(*NA*)^2^*Z* are the normalized radial and axial coordinates of the observation point in the focal zone, respectively. *J*_0_ is Bessel function of the first order. *R* and *Z* are the genuine radial and axial coordinates of the observation point. *NA* is the numerical aperture of the objective lens, *λ* and *w* are the wavelength and relative waist width of incident Gaussian beam. *w*=*w*_0_/*D*, where *D* is the genuine radius of the apodizer and *w*_0_ is the genuine radius of the incident beam [17,20,21]. As *ϕ_j_* is changed from 0° to a certain degree, the intensity of the near-field lobe is increased while the intensity of the far-field lobe (=original single lobe) is decreased. When *ϕ_j_* becomes 180°, the intensity of near- and far-field lobes are almost same, and thus the intensity difference between two lobes becomes minimal.

In this study, the multi-radial beam was generated by using a dual-element transducer with a confocal point. The phase of individual element was controlled by the input signal. Note that this procedure provides similar effect of the phase mask or apodizer in optics. The phase inverted input signals were simultaneously applied to the each element, and thus the focal shift phenomenon was generated.

### 2.2. Sound Field Simulation

An ultrasound field simulation by using Field-II program was conducted to demonstrate the performance of the proposed method. In this simulation, the dual-element aperture was generated by using the procedure to create the aperture consisting of small rectangles, and subsequently, the pulse echo data was obtained. Note that the spatial impulse response of Field-II simulation is calculated through the Tupholme and Stepanishen methodology [22–24].

**Figure 1.**
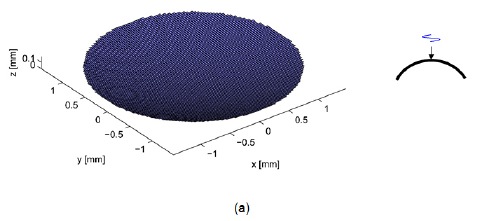

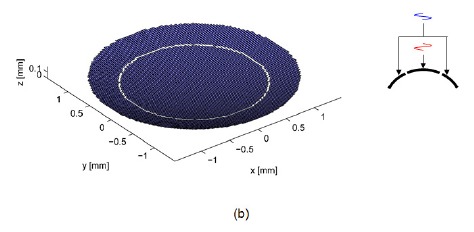
Computed aperture configuration for (**a**) single element transducer and (**b**) dual-element transducer with inverted phase excitation. Right side figures show the applied electrical signals.

Two sets of simulation were performed: a single element and a dual-element transducer with different phase excitation. The simulation result of a single element transducer was used as the reference to evaluate the performance of the dual-element transducer. Disc- and ring-type apertures were created by a function of an arbitrary aperture generation composed of square elements with 100 μm size as shown in Figure 1. The outer diameter is 3 mm and the inner diameter is 2.1 mm considering the absolute intensities of two focal lobes, *i.e.*, the area of the inner element and the outer element are almost similar.

The lateral view width is from −1.5 mm to 1.5 mm, and the axial view depth is from 0.1 mm to 12 mm. The center frequency of all transducers was 40 MHz and the attenuation coefficient for water was about 4 dB/cm at 40 MHz [1]. The overall transmitted beam profile for the phase apodization scheme is simultaneously generated considering the phase interaction. Figure 1 shows the configuration of the single element and the dual-element transducer with phase inversion. Simulation parameters are summarized in Table 1.

## 3. Results

**Figure 2.**
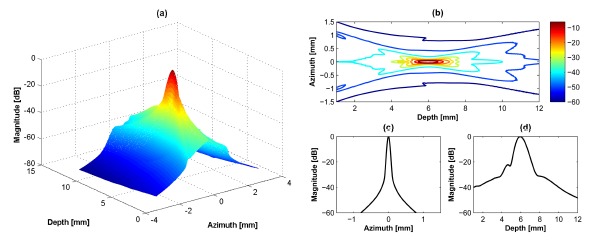
Simulation about single element transducer: (**a**) 3D transmit beam profile, (**b**) 2D transmit beam distribution, (**c**) lateral beam plot, and (**d**) axial beam plot. Note that the color bar in (b) indicates the intensity value in decibel scale and applied to (a). (c) and (d) are obtained from the maximal intensity at 6 mm focal point. The position of the transducer is on the azimuth direction in (a).

**Figure 3.**
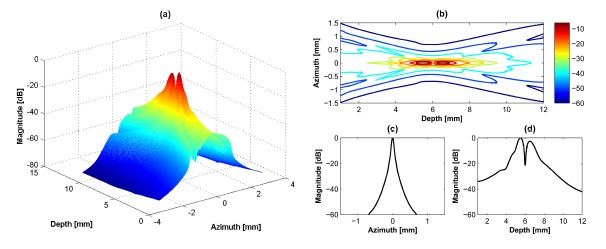
Simulation about dual-element transducer with phase-inverted excitation: (**a**) 3D transmit beam profile, (**b**) 2D transmit beam distribution, (**c**) lateral beam plot, and (**d**) axial beam plot. Note that the color bar in (b) indicates the intensity value in decibel scale and applied to (a). (c) and (d) are obtained from the maximal intensity at the first focal lobe (5.5 mm). The position of the transducer is on the azimuth direction in (a).

A 40 MHz single element transducer generates a single focal point in the axial direction as shown in Figure 2. The –6 dB lateral beamwidth at 6 mm and the −6 dB DOF are 0.08 mm and 1.0 mm, respectively. In the case of the dual-element transducer with phase inversion, two focal zones are simultaneously generated in the axial direction as shown in Figure 3. The total −6 dB DOF by summing of DOFs in the near- and far-lobes is 1.4 mm which is 40% broader than the single element transducer. At the first focal lobe (5.5 mm), the −6 dB lateral beamwidth is 0.08 mm and this is similar to that of the single element transducer, but the −20 dB lateral beamwidth is 0.2 mm, which is 0.04 mm longer than the single element transducer resulting in slightly reduced spatial resolution. In Figure 3d, there is a low intensity part at focal point due to abrupt change of intensity between two peaks. This artifact may be not critical considering the results of point and cyst phantom simulations. Table 2 shows the summarized simulation results.

**Figure 4.**
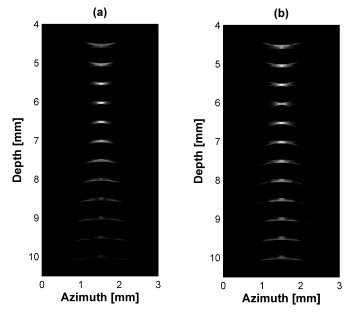
Point target simulation results for (**a**) single element and (**b**) dual-element transducer with phase inversion. All figures are logarithmically compressed with a dynamic range of 60 dB.

Figure 4 shows the point target simulation results using Field II program. In all cases, the aperture size is 3 mm diameter and focal depth is 6 mm. Figure 4a displays simulated point targets using a conventional single element transducer and (b) a dual-element transducer. In Figure 4b, there is a low intensity part at focal point due to abrupt change of intensity between two peaks.

**Figure 5.**
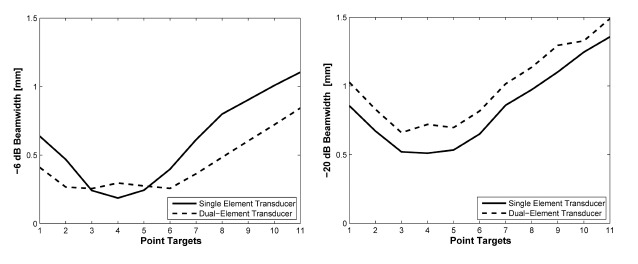
Plot of −6 dB and −20 dB lateral beamwidth throughout the depth of the image in Figure 4.

**Figure 6.**
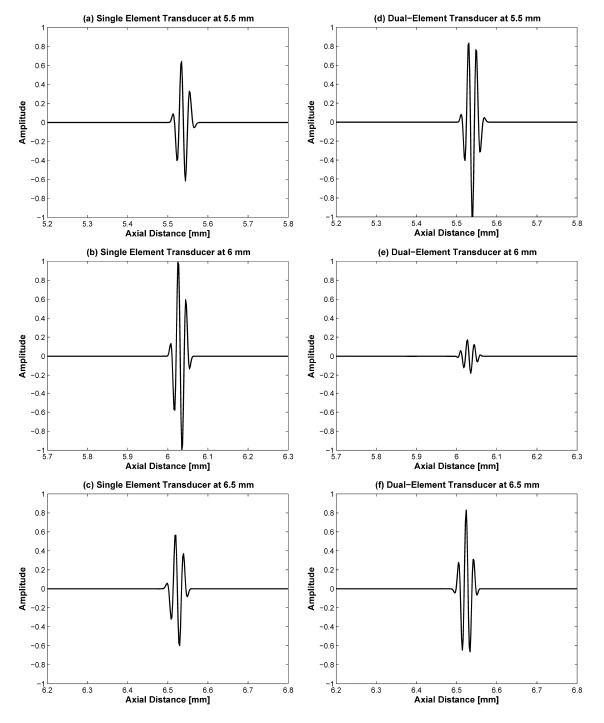
Comparison of the pulse shape between single and dual-element transducers around the focal region.

Figure 5 shows the plots of −6 dB and −20 dB lateral beamwidth throughout the depth of the point targets in Figure 4. The −6 dB lateral beamwidth of the dual-element transducer with phase inversion is better than that of the single element transducer due to the effect of dual focusing at 3rd and 5th targets. However, the −20 dB lateral beamwidth is lower than the single element. At the 4th target in the focal point, the −6 dB lateral beamwidth is higher than single element transducer due to phase distortion. In Figure 6 and Table 3, the −20 dB axial beamwidth of the single element transducer around the focal region is about 6% broader than that of the dual-element transducer with phase inversion. Note that there is phase inversion around focal point in the proposed method as shown in Figure 6.

**Figure 7.**
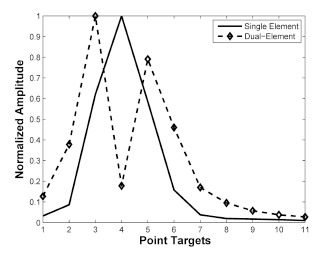
Normalized amplitudes of two cases: (Solid line) single-element transducer, (Dashed line with diamonds) dual-element transducer. Note that the depth of field (DOF) and signal to noise ratio (SNR) of the proposed method are improved compared to single-element mode.

Figure 7 shows the normalized intensity amplitude of the point targets in Figure 4. Normalization was conducted by dividing the maximal value of each B-mode image. In each mode, SNR difference between focal zone and near- and far-field zone is important in ultrasound imaging. The single element transducer had a peak value at 4th target at 6 mm focal depth. However, the relative SNR difference between focal depth and other fields was high. The dual-element transducer shows two peaks at 3rd and 5th targets and the relative SNR difference was small and thus results that more targets in the near- and far- field were visibly detected.

**Figure 8.**
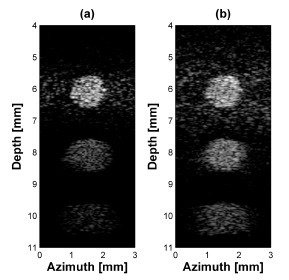
Cyst target simulation results for (**a**) single element and (**b**) dual-element transducer with phase inversion. All figures are logarithmically compressed with a dynamic range of 40 dB.

In the case of cyst phantom simulation by using the dual-element transducer (Figure 8), the SNR in the far field target was higher than single element transducer.

## 4. Discussions and Conclusions

In order to extend DOF in HFUI, the feasibility of the phase inversion scheme by using the dual-element transducer was numerically investigated. When two independent signals with the 180° phase difference were applied to a dual-element transducer, a multi-focal zone was generated in the axial direction.

The simulation results show that the −6 dB DOF of the proposed method was 40% broader than the single element transducer. The −6 dB lateral beamwidth was similar to that of the single element transducer but the −20 dB lateral beamwidth was 25% broader than single element transducer. The axial beamwidth of the proposed method is about 6% higher than the single element transducer resulting in slightly reduced spatial resolution.

Through point and cyst phantom simulation, the SNR comparison within the identical mode was conducted. In the proposed method, the SNR difference between focal point and near- and far-field region is smaller than single element transducer. There was a low intensity portion at the focal point; however, it was not critical based on point and cyst phantom simulation results.

These preliminary simulation results show that the phase inversion scheme combined with a dual-element transducer may generate multiple foci in the axial direction resulting in an extended DOF. The proposed technique can increase SNR, especially in the near- and far-field. This effect will significantly improve the image quality of HFUI. It is important to obtain good resolution in the region of interest but it is also important to achieve a larger bright area in the ultrasound images. This is more critical in the high frequency ultrasound imaging due to high attenuation. In the proposed method, the improved SNR about two times beyond the focal depth will help to monitor deeper targets although the spatial resolution is slightly reduced compared to the single element transducer. Additionally, since the proposed method can improve the length of DOF without complicated hardware system, this technique can be applied to the high frequency ultrasound imaging system with a single element transducer.

There will be several design considerations such as alignment, electrical impedance, piezoelectric material for each element when the proposed method is implemented by the prototype transducer. Thus, more careful design and fabrication process will be required compared to the conventional single element transducer. The performance of the proposed technique will be further evaluated in future work by fabricating a prototype dual-element transducer.

## Figures and Tables

**Table 1.** Simulation parameters for the single and the dual-element transducer.

	**Single Element Transducer**	**Dual-Element Transducer**	

		**Inner-Disc Type**	**Outer-Ring Type**
Frequency [MHz]	40	40	40
−6 dB Bandwidth [%]	65	65	65
Inner Diameter [mm]	–	–	2.1
Outer Diameter [mm]	3	2.1	3
Focal Depth [mm]	6	6	6
F-number	2	3	2

**Table 2.** Simulation results for the single element and dual-element transducers.

	**Single Element Transducer**	**Dual-Element Transducer**
−6 dB DOF [mm]	1.0	1.4
−6 dB Lateral Beamwidth [mm]	0.08	0.08
−20 dB Lateral Beamwidth [mm]	0.16	0.2

**Table 3.** Comparison of −20 dB axial beamwidth between single and dual-element transducers in Figure 6.

	**Position of Targets**		

	5.5 mm	6.0 mm	6.5 mm
Single Element Transducer	50.4 μm	50.5 μm	53.1 μm
Dual-Element Transducer	54.7 μm	54.9 μm	53.2 μm
